# Rapid Classification of Milk Using a Cost-Effective Near Infrared Spectroscopy Device and Variable Cluster–Support Vector Machine (VC-SVM) Hybrid Models

**DOI:** 10.3390/foods13203279

**Published:** 2024-10-16

**Authors:** Eleonora Buoio, Valentina Colombo, Elena Ighina, Francesco Tangorra

**Affiliations:** 1 Department of Veterinary Medicine and Animal Science, University of Milan, Via dell’Università 6, 26900 Lodi, Italy; eleonora.buoio@unimi.it (E.B.); elena.ighina@unimi.it (E.I.); 2 Federchimica AISA, Via G. da Procida, 11, 20149 Milan, Italy; v.colombo@federchimica.it

**Keywords:** near infrared spectroscopy (NIRS), variable cluster, support vector machine, hybrid model, machine learning, milk classification

## Abstract

Removing fat from whole milk and adding water to milk to increase its volume are among the most common food fraud practices that alter the characteristics of milk. Usually, deviations from the expected fat content can indicate adulteration. Infrared spectroscopy is a commonly used technique for distinguishing pure milk from adulterated milk, even when it comes from different animal species. More recently, portable spectrometers have enabled in situ analysis with analytical performance comparable to that of benchtop instruments. Partial Least Square (PLS) analysis is the most popular tool for developing calibration models, although the increasing availability of portable near infrared spectroscopy (NIRS) has led to the use of alternative supervised techniques, including support vector machine (SVM). The aim of this study was to develop and implement a method based on the combination of a compact and low-cost Fourier Transform near infrared (FT-NIR) spectrometer and variable cluster–support vector machine (VC-SVM) hybrid model for the rapid classification of milk in accordance with EU Regulation EC No. 1308/2013 without any pre-treatment. The results obtained from the external validation of the VC-SVM hybrid model showed a perfect classification capacity (100% sensitivity, 100% specificity, MCC = 1) for the radial basis function (RBF) kernel when used to classify whole vs. not-whole and skimmed vs. not-skimmed milk samples. A strong classification capacity (94.4% sensitivity, 100% specificity, MCC = 0.95) was also achieved in discriminating semi-skimmed vs. not-semi-skimmed milk samples. This approach provides the dairy industry with a practical, simple and efficient solution to quickly identify skimmed, semi-skimmed and whole milk and detect potential fraud.

## 1. Introduction

The composition of raw milk significantly impacts the nutritional, physical, and chemical properties of commercially available milk, affecting both the quality and financial value of the product. Milk adulteration is a fraudulent practice used to increase product volume and reduce production costs, potentially affecting the concentration of the nutrients in milk. In Italy, the dairy industry ranks third in terms of infringements, behind the wine and olive oil sectors. In 2022, the authorities recorded 25 infringements in Italy, resulting in the seizure of more than 344,000 kg of goods worth more than EUR 6.3 million [1].

The dairy industry is plagued by fraudulent practices that increase volume, cause physicochemical changes, or extend the shelf life of the product [2]. Falsifying milk fat content is one of the most common forms of food fraud, as fat is a key nutrient that determines the richness and flavor of milk. The Gerber method is the standard method of determining the fat content of dairy products in Europe. It is relatively simple, but it requires many tools (butyrometer, pipette, centrifuge, water bath) to be carried out; it is time-consuming, cannot be automated, and involves the use of hazardous chemicals (sulfuric acid and amyl alcohol). It is also prone to systematic errors, leading to discrepancies between the milk analyzed and the results obtained. The Babcock method is more common in the United States, and it is similar to the Gerber method in terms of disadvantages [3].

The use of near infrared spectroscopy (NIRS) in milk analysis has become popular for detecting fraud related to milk quality or authenticity. NIRS provides detailed information on milk composition by assessing parameters such as fat, protein, lactose, and non-fat solids. Portable spectrometers have enabled in situ analysis with analytical performance comparable to that of benchtop instruments. Their increasingly smaller size, lower cost and greater robustness make them useful for routine characterization and valuable to the process chain, measuring milk nutrients and identifying adulteration through quality assessment procedures [4]. Infrared spectroscopy is a widely used method for differentiating pure and adulterated milk from different species, including goats [5,6], donkeys [7], and camels [8]. NIRS is used for several milk analyses, including identifying geographical origin, classifying milk, determining animal feeding regimes [9], and promoting the early detection of cow nutritional status and health [9,10,11,12]. Furthermore, this is a simple, affordable and environmentally friendly technique that detects adulteration with melamine, urea, and whey [13]. Some constituents remain difficult to predict with NIRS because they are not associated with specific absorption bands (e.g., minerals) or are present in very low concentrations (e.g., volatile compounds) [14]. Iwamoto et al. [15] reported a sensitivity limit of 0.1% for most constituents.

NIRS works by using the absorption and reflection of light in the wavelength range of 750 to 2500 nm. This spectral region is extremely sensitive to molecular vibrations, allowing a comprehensive analysis of the chemical composition of samples. The strong absorption band of water, which can mask other bands such as fat, protein, and lactose, affects the absorption spectrum of milk, while the effects of hydrogen bonding and sample temperature can affect the reliability of NIRS results [16]. In addition, signal distortion can be caused by fat particles and casein micelles in suspension [9]. Therefore, the NIRS output must be mathematically pre-processed to reduce spectral distortions and minimize confounding influences on the spectral analysis [17]. Chemometrics is used to assist in interpreting the complex vibrational spectra obtained during sample analysis, ensuring more accurate and reliable results. Advanced mathematical algorithms can be used to establish correlations between NIR spectral data and reference chemical information, enabling the creation of predictive models for detecting milk fraud.

Partial Least Squares (PLS) regression is the primary chemometric technique used alongside NIR spectroscopy. PLS regression is a multivariate statistical technique used to model the relationships between predictor variables and a response variable. It is useful when dealing with datasets characterized by high dimensionality, multicollinearity, or noisy variables. Calibrations are generally robust if the calibration set reflects the range of variability expected in unknown samples. The main limitations are that large numbers of samples are required to make accurate calibrations, and collinear constituent concentrations must be avoided [18].

The increasing availability of portable NIRS technology has led to the evaluation of alternative supervised techniques, including support vector machine (SVM). SVM is a supervised learning algorithm originally designed as a binary classifier but later extended to handle multi-class classifications using kernel functions. SVM is often used for its simplicity and flexibility in addressing a range of classification problems. The main advantages are that it is computationally cheaper and can be trained with a smaller number of samples compared to other machine learning classifiers. The selection of the optimal kernel and its parameters is the main challenge [19]. SVM can also be used in conjunction with other methods, such as the hybrid PCA-SVM technique. PCA is a statistical technique for reducing the dimensionality of a dataset by finding a new set of variables or principal components, which are linear combinations of the original features, orthogonal (uncorrelated), and reflect the maximum variance in the data [20]. The combination of PCA and SVM offers significant advantages, such as better generalization by removing noise from the data and higher computational efficiency by reducing the number of features, leading to more robust models and speeding up the training and prediction processes of the SVM. To ensure accuracy, the classifier relies on the principal components obtained by PCA rather than the spectra. Discriminant analyses on various products such as honey [21], tea [22], and persimmon varieties [23] have demonstrated high accuracy levels, exceeding 90.6% with PCA-SVM using the radial kernel function. Recently, PCA-SVM models combined with Fourier Transform mid-infrared (FT-MIR) spectroscopy have been investigated as a fast and non-destructive method of classifying milk according to its geographical origin, achieving a classification rate close to 95% [24]. However, PCA has two major issues: all the original features are required to obtain the principal components, and these are much harder to interpret than the original data. 

Unlike PCA, clustering guarantees higher interpretability because it uses original features. By choosing fewer but more important features and eliminating redundant ones, models can be faster and perform better, as they are more effective and less likely to overfit to noise. A simpler model with fewer features is also easier to understand.

Based on these considerations, this paper proposes a novel method based on a compact and low-cost Fourier Transform near infrared (FT-NIR) spectrometer (NeoSpectra-Si-Ware, Cairo, Egypt) [25] coupled with variable cluster–support vector machine (VC-SVM) hybrid models for the rapid classification of milk in accordance with EU Regulation EC N. 1308/2013 [26] without performing any chemical or physical pre-processing.

## 2. Materials and Methods

### 2.1. Milk Samples

In this study, 73 packs of ultra-high-temperature (UHT) treated cow’s milk from various geographic regions, including Italy, France, Austria, the Czech Republic, and Belgium, were purchased from supermarkets. All the samples were in liquid form, and 64 of them complied with EU legislation [26], which requires a fat content below 0.5% (m/m) for skimmed milk, between 1.5% (m/m) and 1.8% (m/m) for semi-skimmed milk, and more than 3.5% (m/m) for whole milk. The remaining nine milk samples had a fat content other than that required by the above-mentioned regulation, and they were labeled as “no-class”. They were used to incorporate more variability. A total of 74% of the milk samples were of Italian origin, and the remaining 26% came from other EU countries. Moreover, of all the samples, 16% came from mountain areas, 5% from organic farms, 19% were lactose-free, and 16% were microfiltered.

The nutritional characteristics of the samples were extracted from the production labels, which contained mandatory details from Regulation (EU) 1169/2011 [27] and sector-specific regulations. The fat content on the label was taken as a reference value to build multivariate prediction models.

Fat, carbohydrate, and protein concentrations were tested for normality using the Shapiro–Wilk test. Since the data did not always show a normal distribution, non-parametric Kruskal–Wallis tests were used for group comparisons, followed by the Steel–Dwass test for multiple comparisons. Significance was forest at *p* < 0.05.

### 2.2. NIR Spectra Acquisition

Milk samples were scanned using the compact and low-cost FT-NIR spectrometer NeoSpectra (Si-Ware, Cairo, Egypt) [25]. The NeoSpectra Micro Development Kit (Si-Ware, Cairo, Egypt) consisted of three tungsten halogen lamps, a monolithic micro-electro-mechanical system (MEMS) Michelson interferometer, and a single InGaAs photodetector. The NeoSpectra Micro was connected to a Raspberry Pi (Raspberry Pi LTD, Cambridge, UK) that acted as a host and allowed connection via universal serial bus (USB) to a laptop. The software (Windows and Linux) allowed the following parameters to be set: scan time, run mode (single or continuous) and data interpolation in each spectrum collected. 

The wavelength range was from 1350 to 2558 nm, and the resolution was set to 16 nm (measured at 1550 nm).

The spectrometer was left to warm up for 20 min before running the analysis in continuous mode. Prior to the first measurement, a background measurement was collected using a Spectralon (99% reflectance). Scanning time was set at 5 s. Milk samples were at room temperature (20 °C) and scanned from the bottom of a Petri dish (90 mm diameter, 15 mm high), filled with approximately 25 g, in reflectance mode for wavelengths of 1350 to 2558 nm. A total of 20 spectra were collected for each sample (5 scans for each quarter rotation of the Petri dish).

### 2.3. NIR Spectra Pre-Treatment

Before building the classification model, the raw NIR spectra underwent several pre-processing techniques to remove irrelevant information [28,29] that could negatively affect the model’s performance. The following techniques were evaluated: (i) standard normal variate (SNV), for scatter correction; (ii) Savitzky–Golay first and second derivatives, for smoothing raw spectra and enhancing the resolution of overlapping peaks.

The best technique was revealed to be SNV, which is commonly used to remove scattering effects from a measured spectrum [30]. Consequently, all spectra used in the classification model were pre-treated with SNV before further analysis.

### 2.4. Cluster-SVM Hybrid Classification Models

Multivariate data analysis was performed using the statistical software JMP Pro 17.2 (SAS Institute, Cary, NC, USA) with the spectral tools developed by Worley [31]. The dataset was randomly divided into two subsets: the training set consisted of 50% of the milk samples, while the validation set comprised the remaining half. Data analysis was performed using the average of twenty spectra recorded for each milk sample.

Using the Cluster Variables platform of JMP Pro 17.2, the entire set of variables was partitioned into six clusters, and, for each cluster, a cluster component was constructed using the first principal component of the variables in that cluster. The six cluster components obtained, representing 97.5% of the variation in the dataset, were used as the input for the support vector machine (SVM).

An SVM model is a supervised learning algorithm used to predict new observations. Based on the training data where the responses are known, the algorithm (kernel function) tries to find the optimal hyperplane that can be used to classify new data points. The most commonly used kernels include the linear kernel and the radial basis function (RBF), which create, respectively, a linear and a nonlinear hyperplane to separate the classes. The goal with an SVM is to fit a model such that the error between a predicted response and the actual response falls within a range of −ε to ε (insensitive region). The penalty associated with misclassifying an observation in the training set represents the cost parameter (C). A higher C parameter implements an algorithm less likely to misclassify a point in the training set, whereas a lower C parameter produces a wider margin. SVM model performance depends on selecting the kernel and setting the parameters C and ε. Reducing ε usually increases the size of C [32]. In addition to the C parameter, the RBF kernel defines the Gamma parameter (γ) that determines the amount of curvature there is to the decision line. A higher Gamma value indicates more curvature. Both the linear and RBF kernels were tested using minimum and maximum values for the parameters ε, C, and γ, as well as the default JMP Pro 17.2 statistical software values (ε = 0.1; C = 0.01 to 5; γ = 0.001 to 0.5), and repeating the procedure 20 times.

The best-performing algorithm was identified based on the highest accuracy rate, calculated as the number of proper classifications divided by the total number of observations. Multi-class confusion matrices were used for this purpose. To further evaluate the classification performance of the algorithms, the confusion matrices were converted into a one-vs.-all type matrix (binary-class confusion matrix) to apply the following class-wise metrics based on the number of true positives (TPs), false positives (FPs), and false negatives (FNs) from both dimensions of the matrix, with the remaining numbers contributing to the true negatives (TNs) [33]:

- Sensitivity, which measures the proportion of the positive responses correctly identified as positive by the classifier:

$$\frac{TP}{TP+FN}$$

- Specificity, which measures the proportion of the negative responses correctly identified as negative by the classifier:

$$\frac{TN}{TN+FP}$$

- MCC (Matthew’s correlation coefficient):

$$\frac{\left(TP\times TN\right)-\left(FP\times FN\right)}{\sqrt{\left(TP+FP\right)\times \left(TP+FN\right)\times \left(TN+FP\right)\times \left(TN+FN\right)}}$$

The range of this metric is from −1 to +1. In the case of an MCC of 1, it can be assumed that FPs and FNs are equal to 0, whereas, in the case of an MCC of −1, the classifier always misclassifies with TPs and TNs equal to 0. As a rule of thumb, values of 0.70 or higher and −0.70 or lower indicate a strong positive or negative relationship, respectively.

## 3. Results and Discussion

Table 1 shows the descriptive statistics for fat, carbohydrate, and protein variables. As expected, statistically significant differences (*p* < 0.01) were found for fat. At the same time, no trends were observed for the other milk characteristics such as protein and carbohydrate content, milk treatments (e.g., lactose-free, filtration), farm management (conventional, organic), geographical area (plain, mountain) or country of origin (Italy, other EU countries). This is consistent with the results of Riu et al. [34] in a study aimed at assessing the use of portable NIR spectroscopy for the rapid and cost-effective analysis of milk without pre-treatments.

**Figure 1:**
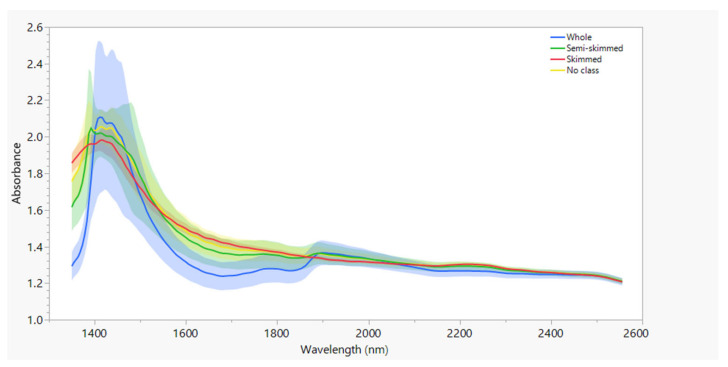
Mean absorbance spectra (solid line) and variation between the mean minus one standard deviation and mean plus one standard deviation of all spectra (shaded areas).

**Figure 2:**
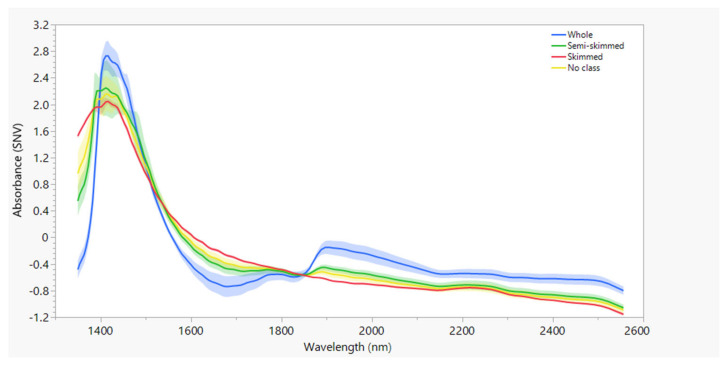
Mean absorbance spectrum (solid line) after being processed with SNV and variation between mean minus one standard deviation and mean plus one standard deviation of all spectra (shaded areas).

The mean raw absorbance spectra and mean absorbance spectra after SNV are shown in Figure 1 and Figure 2. The spectra are colored by fat content according to the classification in the European Regulation: blue, green, and red for whole, semi-skimmed, and whole milk, respectively. Milk with no class is colored yellow. Compared to the raw spectra (Figure 1), SVN (Figure 2) reduced the variation as measured by the standard deviation (shaded areas). The main broad peak at around 1450 nm for all the milk classes was due to the high water absorption. Although several absorption bands corresponding to major milk constituents have been reported in the 1350–2558 nm wavelength range [35,36], the characteristic absorption bands of the milk constituents are very weak and difficult to visualize compared to the water band.

The linear kernel SVM and the RBF kernel SVM performed similarly, showing an overall accuracy rate of 94.4%. This indicates that both models correctly classified 94.4% of the samples in the validation dataset. The accuracy in predicting each milk class was 100% for the whole, semi-skimmed, and skimmed milk samples, while 50% of the no-class milk samples was wrongly classified as semi-skimmed and skimmed milk samples (Table 2 and Table 3).

Table 4 and Table 5 show the converted one-vs.-all confusion matrices for each class of milk (whole, no-class, semi-skimmed and skimmed) for the training and validation sets when the linear kernel and the RBF kernel classifiers were used, respectively. The classification performance of both kernels, based on the class-wise metrics (sensitivity, specificity, MCC) applied to the one-vs.-all type matrix (binary-class confusion matrix) for the training and validation sets, are shown in Table 6 and Table 7.

In the validation dataset, the linear kernel SVM correctly classified the whole and not-whole milk samples (100% sensitivity, 100% specificity, MCC = 1). A sensitivity of 100% was also achieved in discriminating no-class milk samples. In comparison, the specificity for the not no-class milk samples was 94.1%, indicating that the model correctly classified 32 out of 34 (94.1%) actual not no-class milk samples. The sensitivity of the classifier for the semi-skimmed and skimmed milk samples was 94.4% and 83.3%, respectively, meaning that the model correctly classified 17 out of 18 (94.4%) and 5 out of 6 (83.3%) actual semi-skimmed and skimmed milk samples, respectively. A specificity of 100% was achieved for the classification of the not-semi-skimmed and not-skimmed milk samples, indicating that the classifier correctly identified all these milk samples. The MCC values of 0.97 and 0.90 obtained for the linear kernel SVM when used to discriminate the semi-skimmed vs. not-semi-skimmed and the skimmed vs. not-skimmed milk samples, respectively, indicate a strong correlation between the predicted and actual labels. The lowest MCC value (0.69) obtained for the linear kernel SVM when used to classify the no-class vs. not-no-class milk samples could be due to the low number of milk samples labeled as no-class.

In the validation dataset, the RBF kernel SVM correctly classified both the whole and not-whole milk samples and the skimmed and not-skimmed milk samples (100% sensitivity, 100% specificity, MCC = 1). Similar to the linear kernel SVM, sensitivities of 100% and 94.4% were achieved in discriminating the no-class milk samples and semi-skimmed milk samples, respectively, indicating that the model could correctly classify 100% of the actual no-class milk samples and 17 out of 18 (94.4%) actual semi-skimmed milk samples. Specificities of 94.1% and 100% wee achieved for the classification of the not-no-class and not-semi-skimmed milk samples, respectively, indicating that the classifier correctly identified 32 out of 34 (94.1%) actual not-no-class and all the not-semi-skimmed milk samples, respectively. A perfect correlation (MCC = 1) and a strong correlation (MCC = 0.95) between the predicted and actual labels were obtained for the RBF kernel SVM when used to classify the whole vs. not-whole and skimmed vs. not-skimmed milk samples and the semi-skimmed vs. not-semi-skimmed milk samples, respectively. Also, in this case, the lowest MCC value (0.69) obtained for the RBF kernel SVM when used to classify the no-class vs. not-no-class milk samples could be due to the low number of milk samples labelled as no-class.

Overall, these results confirm the suitability of radial kernel SVM methods combined with portable NIR spectroscopy for discriminating milk samples based on fat content as an alternative to other chemometric methods used for milk classification [34,35]. This protocol could be used for routine quality control applications in the dairy industry, including the on-farm monitoring of milk composition. Recent studies [36,37] have shown that NIR spectroscopy has the potential for estimating the fatty acid content of milk, offering new opportunities such as the real-time monitoring of dairy processes but also challenges in developing advanced calibration models. Unlike traditional analytical methods (mass spectroscopy and gas chromatography), NIR instruments, which have no moving parts, can be connected to Internet-of-Things (IoT) applications and tools. In this way, spectra can be sent to the cloud for remote processing [4].

## 4. Conclusions

This study demonstrated the feasibility of using portable NIR spectroscopy and machine learning algorithms to rapidly classify milk in accordance with EU Regulation EC N. 1308/2013. The results obtained from the external validation of the VC-SVM hybrid models showed a perfect classification capacity (100% sensitivity, 100% specificity, MCC = 1) for the RBF kernel when used to classify the whole vs. not whole and skimmed vs. not-skimmed milk samples. A strong classification capacity (94.4% sensitivity, 100% specificity, MCC = 0.95) was also achieved for discriminating the semi-skimmed vs. not-semi-skimmed milk samples. This approach offers the dairy industry a practical, simple, and efficient solution to quickly identify skimmed, semi-skimmed, and whole milk and detect potential fraud.

## Figures and Tables

**Table 1:** Descriptive statistics for the milk type characteristics (values reported as mean ± std. deviation).

Type of Milk	Milk Samples (*n*)	Fat (%)	Carbohydrates (%)	Proteins (%)
No class	9	0.98 ± 0.12 ^A^	5.01 ± 0.34	3.26 ± 0.16
Skimmed	11	0.17 ± 0.18 ^B^	4.95 ± 0.10	3.40 ± 0.20
Semi-skimmed	34	1.59 ± 0.04 ^C^	4.95 ± 0.09	3.36 ± 0.31
Whole	19	3.71 ± 0.35 ^D^	4.88 ± 0.10	3.35 ± 0.24

^A,B,C,D^ Values in the same column with different superscripts differ significantly (*p* < 0.01).

**Table 2:** Confusion matrices of milk-type classification using NIRS combined with VC-SVM (linear kernel).

Actual	Training Set (*n* = 37) Predicted				Overall Accuracy	Validation Set (*n* = 36) Predicted				Overall Accuracy
	Whole	No-Class	Semi-skimmed	Skimmed		Whole	No-Class	Semi-skimmed	Skimmed	
Whole	9 (100%)	0	0	0	100%	10 (100%)	0	0	0	94.4%
No-class	0	5 (100%)	0	0		0	2 (50.0%)	1	1	
Semi-skimmed	0	0	17 (100%)	0		0	0	17 (100%)	0	
Skimmed	0	0	0	6 (100%)		0	0	0	5 (100%)	

( ) Accuracy in prediction for each milk class.

**Table 3:** Confusion matrices of milk type classification using NIRS combined with VC-SVM (RBF kernel).

Actual	Training Set (*n* = 37)Predicted				Overall Accuracy	Validation Set (*n* = 36)Predicted				Overall Accuracy
	Whole	No-Class	Semi-skimmed	Skimmed		Whole	No-Class	Semi-skimmed	Skimmed	
Whole	9 (100%)	0	0	0	97.2%	10 (100%)	0	0	0	94.4%
No-class	0	4 (80.0%)	1	0		0	2 (50.0%)	1	1	
Semi-skimmed	0	0	17 (100%)	0		0	0	17 (100%)	0	
Skimmed	0	0	0	6 (100%)		0	0	0	5 (100%)	

( ) Accuracy in prediction for each milk class.

**Table 4:** One-vs.-all confusion matrices for each class of milk (linear kernel).

Actual	Training Set (*n* = 37)		Actual	Validation Set (*n* = 36)	
	Predicted			Predicted	
	Whole	Not Whole		Whole	Not Whole
Whole	9	0	Whole	10	0
Not Whole	0	28	Not Whole	0	26
	No-class	Not No-class		No-class	Not No-class
No-class	5	0	No-class	2	0
Not No-class	0	32	Not No-class	2	32
	Semi-Skimmed	Not Semi-Skimmed		Semi-Skimmed	Not Semi-Skimmed
Semi-Skimmed	17	0	Semi-Skimmed	17	1
Not Semi-Skimmed	0	20	Not Semi-Skimmed	0	18
	Skimmed	Not Skimmed		Skimmed	Not Skimmed
Skimmed	6	0	Skimmed	5	1
Not Skimmed	0	31	Not Skimmed	0	30

**Table 5:** One-vs.-all confusion matrices for each class of milk (RBF kernel).

Actual	Training Set (*n* = 37)		Actual	Validation Set (*n* = 36)	
	Predicted			Predicted	
	Whole	Not Whole		Whole	Not Whole
Whole	9	0	Whole	10	0
Not Whole	0	28	Not Whole	0	26
	No-class	Not No-class		No-class	Not No-class
No-class	3	0	No-class	2	0
Not No-class	2	32	Not No-class	2	32
	Semi-Skimmed	Not Semi-Skimmed		Semi-Skimmed	Not Semi-Skimmed
Semi-Skimmed	17	1	Semi-Skimmed	17	1
Not Semi-Skimmed	0	19	Not Semi-Skimmed	0	18
	Skimmed	Not Skimmed		Skimmed	Not Skimmed
Skimmed	6	0	Skimmed	5	0
Not Skimmed	0	31	Not Skimmed	0	31

**Table 6:** Classification performance of the linear kernel for each milk class.

Milk Class	Training Set (*n* = 37)			Validation Set (*n* = 36)		
	Sensitivity (%)	Specificity (%)	MCC	Sensitivity (%)	Specificity (%)	MCC
Whole	100	100	1	100	100	1
No class	100	100	1	100	94.1	0.69
Semi-skimmed	100	100	1	94.4	100	0.97
Skimmed	100	100	1	83.3	100	0.90

**Table 7:** Classification performance of the RBF kernel for each milk class.

Milk Class	Training Set (*n* = 37)			Validation Set (*n* = 36)		
	Sensitivity (%)	Specificity (%)	MCC	Sensitivity (%)	Specificity (%)	MCC
Whole	100	100	1	100	100	1
No-class	100	94.1	0.75	100	94.1	0.69
Semi-skimmed	94.4	100	0.95	94.4	100	0.95
Skimmed	100	100	1	100	100	1

## Data Availability

The original contributions presented in the study are included in the article, further inquiries can be directed to the corresponding author.
